# Nonclinical Exon Skipping Studies with 2′-*O*-Methyl Phosphorothioate Antisense Oligonucleotides in *mdx* and *mdx-utrn−/−* Mice Inspired by Clinical Trial Results

**DOI:** 10.1089/nat.2018.0759

**Published:** 2019-03-29

**Authors:** Maaike van Putten, Christa Tanganyika-de Winter, Sieto Bosgra, Annemieke Aartsma-Rus

**Affiliations:** ^1^ Department of Human Genetics, Leiden University Medical Center, Leiden, the Netherlands.; ^2^ Biomarin Nederland BV, Leiden, the Netherlands.

**Keywords:** antisense oligonucleotide, exon skipping, dystrophin, mouse model

## Abstract

Duchenne muscular dystrophy is a severe, progressive muscle-wasting disease that is caused by mutations that abolish the production of functional dystrophin protein. The exon skipping approach aims to restore the disrupted dystrophin reading frame, to allow the production of partially functional dystrophins, such as found in the less severe Becker muscular dystrophy. Exon skipping is achieved by antisense oligonucleotides (AONs). Several chemical modifications have been tested in nonclinical and clinical trials. The morpholino phosphorodiamidate oligomer eteplirsen has been approved by the Food and Drug Administration, whereas clinical development with the 2′-*O*-methyl phosphorothioate (2OMePS) AON drisapersen was recently stopped. In this study, we aimed to study various aspects of 2OMePS AONs in nonclinical animal studies. We show that while efficiency of exon skipping restoration is comparable in young and older C57BL/10ScSn-*Dmd^mdx^*/J (*mdx*/BL10) mice, functional improvement was only observed for younger treated mice. Muscle quality did not affect exon skipping efficiency as exon skip and dystrophin levels were similar between *mdx*/BL10 and more severely affected, age-matched D2-*mdx* mice. We further report that treadmill running increases AON uptake and dystrophin levels in *mdx*/BL10 mice. Finally, we show that even low levels of exon skipping and dystrophin restoration are sufficient to significantly increase the survival of *mdx-utrn*−/− mice from 70 to 97 days.

## Introduction

Mutations in the dystrophin-encoding *DMD* gene are associated with both severely progressive Duchenne muscular dystrophy (DMD) and less progressive Becker muscular dystrophy (BMD) [1]. The dystrophin protein has a stabilizing function in skeletal muscle, where it connects F-actin in the cytoskeleton to β-dystroglycan, which in turn is connected to the extracellular matrix [2]. As such, it acts as a shock absorber to prevent myofiber damage during muscle contraction. In DMD, out-of-frame or nonsense mutations cause premature truncation of translation of dystrophin that consequently cannot fulfill its connector function [3]. In BMD patients, by contrast, mutations maintain the reading frame so that internally deleted dystrophins are produced containing the domains for F-actin and β-dystroglycan binding. These dystrophins are partially functional, as underlined by the slower disease progression observed in BMD patients [1,3].

The exon skipping approach is based on the fact that, in theory, most DMD patients have the genetic capacity to produce BMD-like dystrophins [4]. Exon skipping uses antisense oligonucleotides (AONs) targeting specific exons, with the aim to modulate pre-mRNA splicing. AONs are short DNA or RNA analogs that are chemically modified to improve stability and pharmacokinetic properties. Upon binding of an AON to its target, the exon is hidden from the splicing machinery and skipped from the mRNA. Exon skipping can restore the reading frame of out-of-frame deletions involving one or more exons [4]. For example, an out-of-frame deletion of exon 48–50 can be reframed by exon 51 skipping, while a deletion of exon 45 requires exon 44 skipping. Alternatively, skipping an in-frame exon containing a small mutation will bypass the mutation without affecting the reading frame [4]. Exemplary here is the *mdx* mouse, which has a spontaneous nonsense mutation in exon 23 of the murine *Dmd* gene. Because exon 23 is in-frame, exon 23 skipping can correct dystrophin expression in this model [5,6].

Exon skipping is a mutation-specific approach, since the target exon depends on the size and location of the mutation [7]. Given that most patients have a deletion involving one or more exons and that these deletions cluster between exon 45 and 55, skipping of some exons applies to larger groups of patients, for example, skipping of exon 51, 53, 45, and 44 would apply to 13%–14%, 8%, 8%, and 6% of patients, respectively [7,8].

Initial exon skipping studies involved primary cell cultures derived from the *mdx* mouse and DMD patients carrying various deletions, duplications and small mutations and 2′-*O*-methyl phosphorothioate (2OMePS) AONs [6,9–12]. The *in vitro* studies provided proof-of-concept that 2OMePS AONs targeting various exons could indeed induce exon skipping and dystrophin restoration. *In vivo* studies focused initially on two chemical AON modifications: the aforementioned 2OMePS and the phosphorodiamidate morpholino oligomer (PMO) [5,13–18].

From a pharmacokinetic perspective these modifications are vastly different. The 2OMePS AON is negatively charged. Furthermore, the phosphorothioate (PS) backbone enables low affinity binding to serum proteins, which reduces renal clearance and increases plasma half-life, to prolong the window for tissue uptake [19]. However, this advantage comes at a cost. Side effects reported for AONs with a PS backbone include injection site reactions upon subcutaneous injection, complement activation, thrombocytopenia, and an inflammatory response, although the latter is sequence motif dependent. PMOs, by contrast, are uncharged, and serum protein binding is low [20]. Consequently, they are rapidly cleared by the kidney. However, the short residence time can be compensated for by higher dosage, given that PMOs appear to be relatively inert: no major safety events have been observed until date.

Both 2OMePS and PMOs targeting exon 23 in the *mdx* mice were able to induce exon 23 skipping and dystrophin restoration in skeletal muscle upon local and systemic treatment [6,13–18,21]. Notably, it was shown that upon intravenous injection of 2OMePS AON, uptake and exon skipping levels were much higher in dystrophic muscle than healthy muscle [17]. Interestingly, after 2OMePS AON treatment, a small increase in dystrophin expression was observed for most fibers, while for PMO treatment dystrophin expression seemed to be patchier, with a subset of fibers expressing high levels of dystrophin [13,15,21–25]. This might be related to the differences in protein-binding kinetics and cellular uptake mechanisms.

For both chemistries, candidates targeting exon 51-eteplirsen (PMO chemistry, developed by AVI-BioPharma/Sarepta) and drisapersen (2OMePS chemistry, developed by Prosensa/GlaxoSmithKline/BioMarin)—were the first to be tested clinically. After intramuscular and short dose-finding tests with eteplirsen [26,27], a subsequent trial tested 30 and 50 mg/kg eteplirsen or placebo in 12 patients [24]. After 24 weeks an open-label study was initiated where all patients received 30 or 50 mg/kg eteplirsen. Muscle biopsies revealed dystrophin restoration after 48 weeks of treatment for both doses [24]. Six-minute walking distance (6MWD) declined over the course of the 188-week follow-up, the decrease was less than in age-matched natural history subjects from Belgium and Sweden [28]. In all subjects, eteplirsen treatment was well tolerated. Dystrophin levels quantified by western blot, revealed an increase to up to 0.9% of healthy controls after 188 weeks of treatment [29]. Meanwhile, Sarepta had initiated a confirmatory phase 3 study and could show an increase to up to 0.4% of healthy controls in some patients after 48 weekly treatments. Based on these data, Food and Drug Administration (FDA) gave eteplirsen accelerated approval in 2016 [29,30]. Evaluation by the European Medicines Agency (EMA) recently resulted in a refusal of the marketing authorization for eteplirsen.

For drisapersen, also first a local injection trial and a dose-finding study were performed [31,32]. The 12 patients involved in the dose-finding trial were enrolled in an open-label study and treated on and off with weekly doses of 6 mg/kg drisapersen for close to 6 years. After 188 weeks, the 6MWD was stabilized for 8/10 patients who were ambulant at the start of the open-label study, while a decline was expected based on natural history data in age-matched DMD patients [33,34].

After the dose escalation study, three double-blind placebo controlled trials were initiated in parallel: a phase 2 study in 51 patients to compare 3 and 6 mg/kg, a phase 2 study in 53 patients to compare different dose regiments and a confirmatory phase 3 study in 186 patients. The phase 2 studies enrolled mainly early stage patients (6–8 years old, rise time from floor below 7 s), whereas the phase 3 study involved both early and later stage patients (5–16 years old). The 6-min walk test was the primary endpoint in each study and as such only ambulatory patients were enrolled.

In all studies side effects were observed, primarily injection site reactions, subclinical proteinuria, and thrombocytopenia in 2% of patients [31,34,35]. On a functional level, there was a nonsignificant trend suggesting an increase in the 6MWD of ∼30–40 m for patients treated with weekly doses of 6 mg/kg compared with placebo for both phase 2 studies [29,35]. However, for the phase 3 study, the difference between the treated and placebo group was only 11 m, which was not statistically significant, nor clinically relevant. *Post-hoc* analysis of the aggregated data revealed that the difference was larger for patients in an early stage of the disease. Based on these data BioMarin applied for approval with the FDA and the EMA. FDA did not grant approval and subsequently the company withdrew the marketing authorization application with EMA and announced to stop the clinical development of drisapersen as well as other compounds in clinical development for exon 44, 45, and 53 skipping, and instead to focus on the nonclinical optimization of AONs [29].

While animal studies are commonly performed in advance of clinical trials, they can also be used in a reverse translational approach to address questions raised in clinical studies. Some of those questions can be addressed better in animal models than in human trials, for example, because mouse models allow studying of multiple skeletal muscles and heart and allow studies to be conducted in a more controlled way. Although several mouse models are available, most nonclinical AON studies have been performed with the *mdx* mouse in a C57BL/10ScSnJ background (*mdx*/BL10). These mice show a mild phenotype, however, speculated to be due to very efficient regeneration. Crossing the *mdx* mouse to a DBA/2J background (D2-*mdx*) results in an atrophic model with higher levels of fibrosis and impaired regeneration [36,37]. Mice lacking both utrophin and dystrophin (*mdx*-*utrn*−/−) have a severe phenotype, with impaired muscle function and severe muscle pathology leading to an average survival of 6–10 weeks [38–40].

The clinical trials with exon 51 targeting AONs raised some questions that we wanted to study further in nonclinical studies in mouse models. There appeared to be an age of intervention effect in patients, where younger patients were more responsive on a functional level than patients in whom the disease was more advanced. We, therefore, in this study, tested AON treatment in young and old *mdx*/BL10 mice, but also in the more severely affected D2-*mdx* model. Second, even though tissue distribution of 2OMePS AONs, being charged and highly protein bound, is not likely flow limited, we wondered whether a vast increase in blood flow to skeletal muscle by physical exercise might improve AON uptake. We, therefore, compared AON treatment in *mdx*/BL10 mice with and without a treadmill running protocol during AON exposure. Finally, we wanted to assess whether low levels of exon skipping and dystrophin restoration as currently seen in clinical trials are sufficient to increase survival in the severe *mdx*-*utrn*−/− mouse model.

## Materials and Methods

### Ethics approval and mouse husbandry

All experiments were approved by the animal experimental commission (DEC) of the Leiden University Medical Center (LUMC) and performed according to Dutch regulation for animal experimentation. Mice were housed with 12-h light/12-h dark cycles in individually ventilated cages at 20.5°C and had access to standard RM3 (SDS, Essex, United Kingdom) chow and water *ad libitum*.

### AON treatment of animals

Mice received injections with a previously described 2OMePS AON targeting exon 23 [41], and were sacrificed 1 week after the last AON injection.

To study the effect of age, the experiment was performed with groups of seven C57Bl/10ScSn-*Dmd^mdx^*/J (*mdx*/BL10) female mice, which were 4 or 16 weeks old at the initiation of the experiment and were treated with 4 weekly subcutaneous injections of 50 mg/kg for 12 weeks with AONs or saline. This route of delivery, frequency, and dosing was the standard at the time when experiments were conducted. Blood was collected before treatment and after 4, 8, and 12 weeks to determine creatine kinase (CK) levels. Functional tests (two- and four-limb hanging tests and forelimb grip strength) were performed as well in week 0, 4, 8, and 12 on different days of the week. Functional tests were performed as previously described [42] in accordance with the TREAT NeuroMuscularDisorders (TREAT-NMD) standard operating procedures.

To study the effect of muscle quality, six 4-week-old female *mdx*/BL10 and six 4-week-old female D2.B10-*Dmd^mdx^*/J (D2-*mdx*) mice were used. They were treated with weekly intravenous injections of 200 mg/kg AON, for a duration of 8 weeks. This route of delivery was chosen since current trials with 2OMePS compounds by WaVe are conducted utilizing this. This prevented us, however, from injecting multiple times per week as this is unfeasible for intravenous injections.

To study the effect of treadmill running, two groups of six 4-week-old female *mdx*/BL10 mice were treated with 3 weekly subcutaneous injections of 65 mg/kg AON for 9 weeks (total dose per week is 200 mg/kg), where one group was subjected to horizontal treadmill running (30 min at 12 m/min) 15 min after each AON injection. In this study, subcutaneous injections were chosen to allow multiple injections per week as a minimally invasive administration, so as not to interfere with the subsequent running. The frequency was reduced to three times per week as this is known to be tolerated by dystrophic mice. Treadmill running was performed according to the TREAT-NMD standard operating procedures, as previously described [29,42].

To study the effect of AON treatment on survival 30 *Utrn^tm1Ked^Dmd^mdx^/*J (*mdx*-*utrn*−/−) mice of both genders were randomized in a saline or AON group. Mice, 1 week of age, were treated with a single subcutaneous injection of 200 mg/kg per week until death occurred or until animals were sacrificed based on prespecified humane endpoints.

For each study, gastrocnemius, quadriceps, tibialis anterior, triceps, diaphragm, heart, kidney, liver, spleen, and brain were isolated after sacrifice to determine AON, exon skip, and dystrophin levels in tissue.

### Assessing AON tissue levels

For measuring the concentration of the AON in plasma and tissue samples a hybridization/ligation enzyme-linked immunosorbent assay method based on an assay previously published was used [43] following adaptations previously described [44]. Briefly, equal tissue amounts were homogenized in 100 mM Tris-HCl, pH 8.5, 200 mM NaCl, 0.2% sodium dodecyl sulfate (SDS), 5 mM ethylenediaminetetraacetic acid and 2 mg/mL proteinase K using MagNa Lyzer green bead tubes in a MagNa Lyzer (Roche Diagnostics, the Netherlands). Samples were diluted 600–6,000 times (muscles), 2,000–60,000 times (kidney, liver, spleen), or 60–600 times (brain) in pooled control *mdx*/BL10 tissue in phosphate-buffered saline (PBS). Calibration curves of the analyzed AON prepared in 60 times pooled control mouse *mdx*/BL10 tissue in PBS were included. All analyses were performed in duplicate.

### RNA isolation and exon skipping analysis

Skeletal and cardiac muscles were homogenized in TRIsure buffer (GC Biotech, the Netherlands) using the MagNA Lyser and 1.4 mm Zirconium Beads Pre-Filled Tubes (OPS Diagnostics). This was followed by chloroform extraction as previously described [44]. For cDNA synthesis, 400 ng of RNA was used in a 20 μL reaction with random hexamer primers and transcriptor reverse transcriptase (Roche Diagnostics) for 45 min at 42°C. For PCR analysis, 1.5 μL of cDNA was incubated with 1.25 U taq polymerase (Roche Diagnostics), 20 pM of primers (reverse primer in exon 24, forward primer in exon 22) and one time supertaq PCR buffer (Enzyme Technologies Ltd.) and amplified for 40 cycles each consisting of an incubation for 30 s at 94°C, 30 s at 60°C, and 30 s at 72°C. Exon skipping levels were semiquantitatively determined as the percentages of the total (wild type and skipped) product with the Agilent 2100 Bioanalyzer.

### Dystrophin analysis

Muscle tissues were homogenized in 1.4 mm Zirconium Beads Pre-Filled Tubes (OPS Diagnostics) with a MagNA Lyser (Roche Diagnostics). Samples were homogenized for 20 s at speed 7,000 (2–5 rounds) in 1 mL 125 mM Tris-HCl (pH 6.8) buffer supplemented with 20% (w/v) SDS. Protein concentrations were determined by the Bicinchoninic Acid Protein Assay Kit (Thermo Fisher Scientific, the Netherlands) using bovine serum albumin as a standard according to the manufacturer's instruction. Western blotting analysis was done as described previously [45], using GTX15277 as a primary antibody for dystrophin (diluted 1:2,000; Gene Tex) and AB72592 as a primary antibody for alpha-actinin (loading control, diluted 1:1,000; Abcam, United Kingdom). Secondary antibodies, IRDye 800 CW (1:5,000, immunoglobulin G; Li-Cor, NE) and IRDye 680TL (1:10,000 Li-Cor), were used for dystrophin and alpha-actinin, respectively. Membranes were analyzed with the Odyssey system and software (Li-Cor).

### Plasma CK and toxicity marker analysis

Blood was collected in lithium–heparin-coated microvettes CB300 (Sarstedt B.V., the Netherlands). CK, urea, alkaline phosphatase, glutamate oxaloacetate transaminase, and glutamate pyruvic transaminase levels were determined using corresponding Reflotron strips (Roche Diagnostics) in the Reflotron Plus machine (Roche Diagnostics).

### Statistical analysis

Two-way analysis of variance analysis with Bonferroni's *post-hoc* test was performed in Prism4 (GraphPad Software, La Jolla, CA) to compare AON tissue and exon skipping levels between groups. Analysis on the AON tissue levels were performed on log-transformed data. Functional performance was compared between the groups using a linear regression model in SPSS 17.0.2 (IBM, Armonk, NY). The Gehan–Breslow–Wilcoxon test was run for the survival analysis in GraphPad. Correlation between dystrophin levels and survival was assessed with Pearson correlation. Results were deemed significantly different when *P* < 0.05 and values are presented as mean ± standard deviation or ±standard error of the mean.

## Results

### Age did not influence exon skipping efficiency in the mdx mouse, but only younger mice improved functionally

**FIG. 1.**
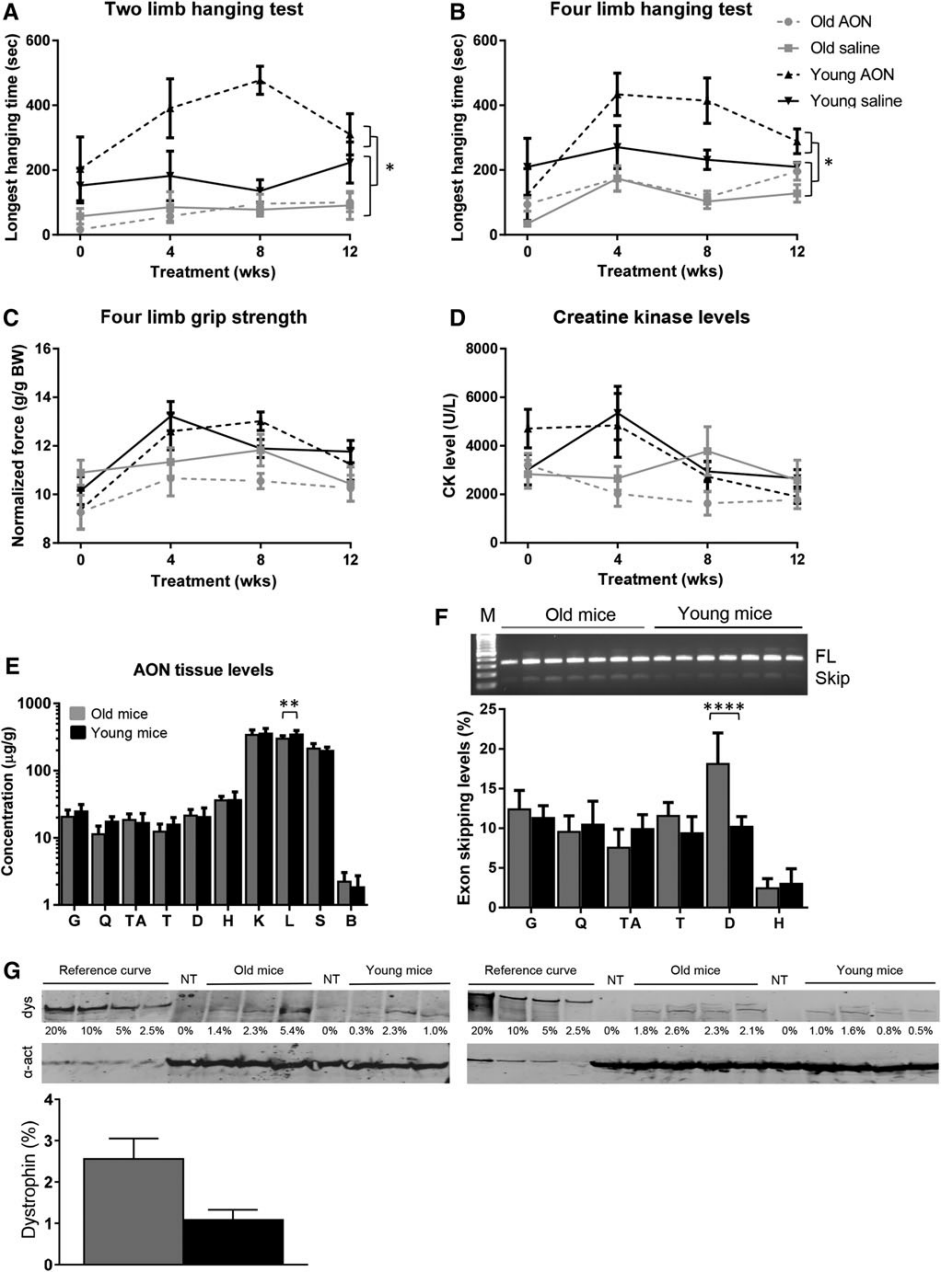
Studying AON treatment in young and old *mdx*/BL10 mice (*n* = 7 per group). **(A)** Two-limb hanging test results. Young mice outperformed older mice. AON treatment improved performance in young, but not in older mice. Wild-type animals are generally able to hang for 10 min (maximum hanging time, not shown). **(B)** Four-limb hanging test results. Young mice outperformed older mice. AON treatment improved performance only in young mice. Wild-type animals are generally able to hang for 10 min (maximum hanging time, not shown). **(C)** Normalized grip strength results. Younger mice tend to be stronger than older mice, but AON treatment did not improve muscle strength. The normalized grip strength of wild-type mice is ∼15–17 g/gBW (data not shown). **(D)** Plasma CK levels. CK levels were similar for the different groups at the start of treatment. For younger mice they decreased with time, whereas this was not observed for older mice. **(E)** AON tissue levels. AON tissue levels were comparable between young and old mice, but only higher in the liver of younger mice. AON levels in skeletal muscles [gastrocnemius (G), quadriceps (Q), tibialis anterior (TA), triceps (T), diaphragm (D), or heart (H) were about 10-fold lower than in kidney (K), liver (L), and spleen (S), while negligible in brain (B)]. **(F)** Exon skipping analysis. Representative agarose image showing RT-PCR fragments of exon 22–24 for the diaphragm (*upper panel*). FL is full-length fragment. Exon skipping levels were quantified by lab-on-a-chip analysis (*lower panel*). No significant differences were observed between young and old mice, except for the diaphragm where exon skipping levels were significantly higher in the old mice. **(G)** Dystrophin levels were quantified using the Odyssey system. The first four lanes contained a concentration series of a wild-type standard. Dystrophin levels of the quadriceps did not largely differ between old and young mice. dys, dystrophin, α-act, α-actinin; NT is nontreated *mdx* (negative control). *P < 0.05, **P < 0.01, ****P < 0.0001. The mean and SEM are provided for **(A**–**D)** and **(G)**, whereas the SD is provided for **(E**, **F)**. AON, antisense oligonucleotide; CK, creatine kinase; g/gBW, gram/gram body weight; RT-PCR, reverse transcription polymerase chain reaction; SD, standard deviation; SEM, standard error of the mean.

To study whether age had an impact on AON uptake and efficiency, we treated groups of seven *mdx*/BL10 mice for 12 weeks with 4 weekly subcutaneous injections of 50 mg/kg 2OMePS AON targeting exon 23 or saline. Treatment was initiated at either 4 or 16 weeks of age. Functional tests were performed before treatment and 4, 8, and 12 weeks after treatment initiation (Fig. 1A–C). In the hanging tests mice had to hang for up to 10 min, where the start position varies between two limbs (wire) and four limbs (grid). Wild-type animals are generally able to hang for 10 min in both tests, whereas *mdx* mice drop off earlier [46]. In our experiments, for both hanging tests, the younger AON-treated mice outperformed all other groups [ie, older saline and AON-treated mice (*P* < 0.003) and the saline-treated young mice (*P* < 0.02)]. AON treatment improved hanging performance in the young mice compared with saline, but mice were still unable to hang for the full 10 min. For the normalized grip strength the younger mice appeared stronger than older mice, but this difference was not significant. AON treatment did not improve grip strength.

Plasma was obtained before treatment, and at 4, 8, and 12 weeks to assess CK levels (Fig. 1D). Increased plasma CK levels are an indication of muscle damage (<500 U/L in wild-type mice). At the start of treatment, CK levels were similar for all groups at ∼4,000 U/L. Levels did not drop upon AON treatment. One week after the last injection, blood was collected before sacrifice to measure kidney and liver function and damage markers. There was no difference observed between AON and saline-treated animals for any of the markers tested and all values were in the normal ranges for *mdx* mice (urea, alkaline phosphatase, glutamate oxaloacetate transaminase, and glutamate pyruvic transaminase, data not shown).

After sacrifice, muscles, kidney, liver, spleen, and brain were isolated for pharmacokinetic analysis (Fig. 1E). No differences in AON levels could be observed between tissues from old and young mice, except for liver, where higher AON levels (*P* < 0.007) were detected in younger mice. As observed previously [17], uptake was highest for kidney, liver, and spleen, about 10-fold lower for skeletal muscle and heart, whereas AON uptake in the brain was virtually absent. RNA was isolated from skeletal muscles and heart and reverse transcription polymerase chain reaction (RT-PCR) analysis was performed to assess exon skipping levels (Fig. 1F). Exon skipping could be confirmed for all AON-treated mice in gastrocnemius, quadriceps, tibialis anterior, triceps, diaphragm, and heart, whereas no exon skipping was observed for saline-treated mice (data not shown). Exon skipping levels were quantified with a lab-on-a-chip analysis revealing similar exon skipping levels for most muscles of young and old mice, except for the diaphragm, where levels in older mice were higher (*P* < 0.0001) than in younger mice. As observed previously [17], exon skipping levels were lower in heart than skeletal muscles. Dystrophin levels assessed by western blot of the quadriceps showed variation, and while levels were higher for older mice, this difference was not significant (average 2.6% ± 1.3% vs. average 1.1% ± 0.7% for old and young mice, respectively) (Fig. 1G).

### Exon skipping efficiency not altered in the more severely affected D2-mdx mouse

**FIG. 2.**
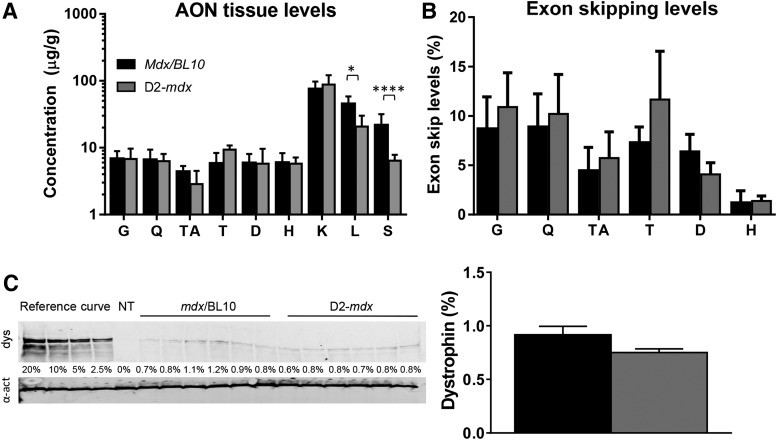
Studying AON treatment in *mdx* mice in a C57/BL10ScSnJ and DBA/2J background (*n* = 6 per group). **(A)** No differences in muscle AON levels were observed between the strains. D2-*mdx* mice had lower AON levels in liver and spleen. **(B)** Exon skipping quantification of RT-PCR fragments by lab-on-a-chip analysis. No significant differences in exon skipping levels were observed between the genetic backgrounds. Gastrocnemius (G), quadriceps (Q), tibialis anterior (TA), triceps (T), diaphragm (D), and heart (H) **(C)**. Western blot analysis of the gastrocnemius muscle. Dystrophin restoration was observed for all treated mice, with no apparent differences between the strains. NT is nontreated *mdx* (negative control). dys, dystrophin; α-act, α-actinin (loading control). *P < 0.05, ****P < 0.0001. The mean and SEM is provided for **(C)**, whereas the SD is provided for **(A**, **B)**. RT-PCR, reverse transcription polymerase chain reaction.

It has been reported that D2-*mdx* mice have higher levels of fibrosis than *mdx*/BL10 mice [36,37]. To study whether the fibrosis impairs AON efficiency (eg, by reduced uptake due to the more extensive fibrosis), we treated groups of six *mdx*/BL10 and six D2-*mdx* mice weekly with intravenous injections of 200 mg/kg AON for 8 weeks. Animals were sacrificed 1 week after the last injection and skeletal muscles, heart, and organs were isolated. AON levels in skeletal muscles did not differ between the two strains (Fig. 2A). D2-*mdx* mice had however lower AON levels in liver and spleen than *mdx*/BL10 mice. RT-PCR analysis revealed exon skipping for all animals for each muscle tested (data not shown). Quantification of exon skipping levels revealed that these were lower in heart than skeletal muscles for both genetic backgrounds (Fig. 2B). There was a tendency for higher exon skipping levels in skeletal muscles for the DBA/2J background. None of these differences reached significance, though. Since the D2-*mdx* muscle has more fibrosis, even similar levels of exon skipping could result in lower levels of dystrophin being produced in these muscles. We, therefore, quantified dystrophin levels by western blot analysis on total protein from the gastrocnemius muscle (Fig. 2C). Low levels of dystrophin were observed for each of the samples, with no apparent difference between backgrounds (0.9% ± 0.2% *mdx*/BL10, 0.8% ± 0.1% D2-*mdx*).

### Treadmill running marginally improved exon skipping efficiency in mice

**FIG. 3.**
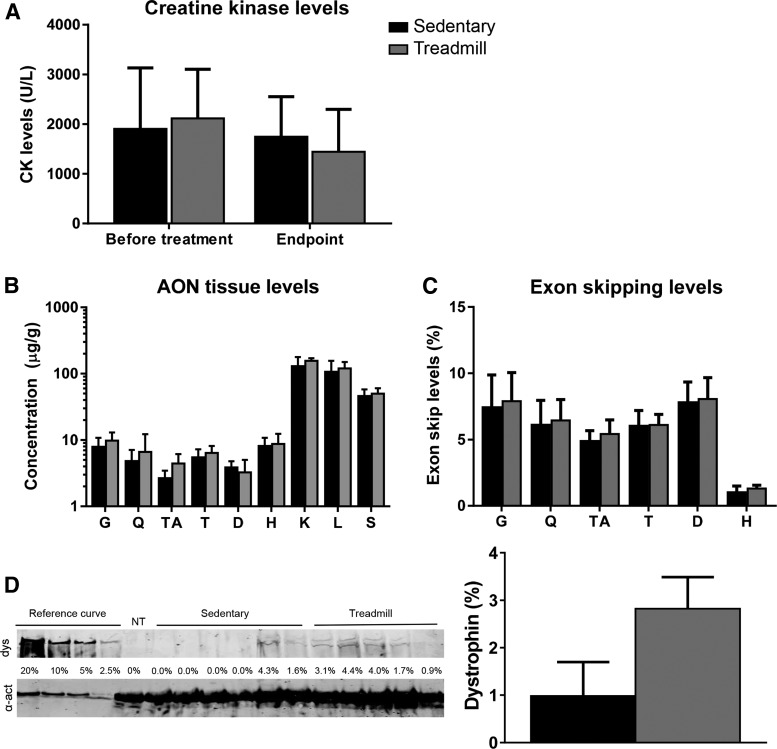
Studying the influence of treadmill running on AON treatment effects (*n* = 6 per group). **(A)** Plasma CK levels. No difference was observed before treatment or at the endpoint. **(B)** AON tissue levels were slightly higher in treadmill-exercised mice. **(C)** Exon skipping quantification of RT-PCR fragments by lab-on-a-chip analysis. Although exon skipping levels in treadmill-exercised mice tended to be higher, the differences were not significant. **(D)** Dystrophin levels of the gastrocnemius assessed by western blot. Dystrophin levels were slightly higher in mice, which were subjected to treadmill running than sedentary mice. NT is nontreated *mdx* (negative control). dys, dystrophin; α-act, α-actinin (loading control). The mean and SEM is provided for **(C, D)**, whereas the SD is provided for **(A**, **B)**.

We speculated that running during AON exposure would improve blood flow to skeletal muscles and thereby increase muscle-specific uptake of AONs and exon skipping levels. We, therefore, treated 12 *mdx*/BL10 mice with 3 weekly subcutaneous injections of 65 mg/kg for 9 weeks. Fifteen minutes after the AON injection, six mice were subjected to treadmill running for 30 min, whereas the other six mice were not. Body weight was captured before each injection. No difference in weight was observed between the treadmill and sedentary groups at any of the time points (data not shown). Plasma CK levels were determined at the start and 1 week after the last AON injection when mice were sacrificed (Fig. 3A). CK levels were similar between the groups before treatment initiation, and at the end of the experiment. Plasma was also used to measure markers for liver and kidney damage and function. Levels for each marker were within the normal range for *mdx*/BL10 mice and there were no differences between the treadmill and the sedentary groups (data not shown).

Muscles were isolated to assess tissue AON levels (Fig. 3B). Overall, AON levels in all tissues, except diaphragm, were higher in mice subjected to treadmill running. This difference reached only significance when all data of each group was combined (*P* < 0.01), but not for individual muscles. Exon skipping levels were determined by RT-PCR and lab-on-a-chip analysis (Fig. 3C). While exon skipping levels in treadmill-exercised mice were higher than in sedentary mice, the increases were minimal and differences were not significant. Dystrophin levels of the gastrocnemius assessed by western blot were slightly higher in mice subjected to treadmill exercise compared with sedentary mice (sedentary 1.0% ± 1.7%, treadmill 2.8% ± 1.5%).

### 2OMePS AON treatment significantly increased survival of mdx-utrn−/− mice

**FIG. 4.**
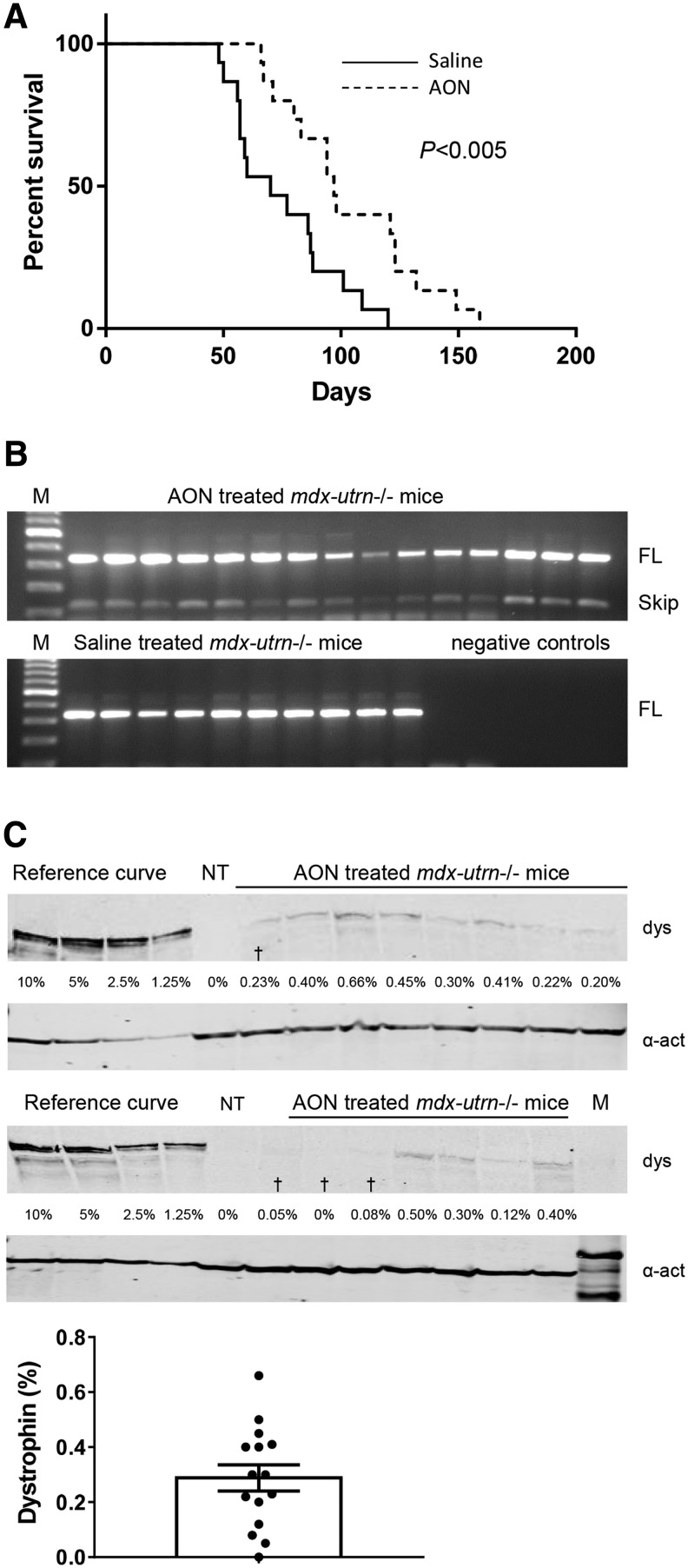
Studying the effect of AON treatment on survival in *mdx-utrn−/−* mice (*n* = 15 per group). **(A)** Survival plot. AON-treated mice lived significantly longer than saline-treated mice. **(B)** RT-PCR analysis. A representative agarose gel image containing RT-PCR fragments of exon 22–24 is shown (gastrocnemius). Exon skipping was only detected for AON-treated mice and not for saline-treated mice. FL is full length fragment **(C)** western blot analysis. Low dystrophin levels were observed in the gastrocnemius of AON-treated mice. *Lanes* indicated with “^†^” include mice which were found dead in their cage despite careful monitoring. NT is nontreated *mdx* (negative control). dys, dystrophin; α-act, α-actinin (loading control). Data are presented as mean ± SEM, whereas *dots* represent individual datapoints.

The exon skipping and dystrophin levels obtained in our experiments are low, but comparable to the levels achieved in clinical trials. Due to the mild phenotype of the *mdx*/BL10 mouse, it is not possible to assess whether these low levels have an impact on survival. The *mdx*-*utrn*−/− mouse is more severely affected and has an average survival of about 8 weeks. However, these mice are very fragile and disease progression starts early in the neonatal stage. To study whether AON treatment had an effect on survival, *mdx*-*utrn*−/− mice were randomized over a saline or AON treatment group, each consisting of 15 mice, which received weekly subcutaneous saline or AON injections at 200 mg/kg/week starting at day 7 of age. Mice were monitored carefully. Animals were sacrificed when they had reached prespecified humane endpoints, consisting of significant weight loss (more than 20% over the course of 3 days), difficulty with breathing or were suffering from severe discomfort. Survival curves reveal that the AON-treated animals lived significantly longer than the saline-treated animals (*P* < 0.005), with median survival of 70 days for saline-treated mice and 97 days for the AON-treated animals (Fig. 4A).

Skeletal muscles were harvested after sacrifice and RNA was isolated for RT-PCR analysis (Fig. 4B). RNA quality was too poor for RT-PCR analysis for five saline-treated mice. Despite careful monitoring these animals were found dead in their cage. RT-PCR analysis was therefore possible for 15 AON-treated and 10 saline-treated mice. Exon skipping could be confirmed in gastrocnemius muscles for each of the treated mice, whereas no exon skipping was observed for saline-treated mice. We refrained from quantifying exon skipping levels or AON tissue levels, since different mice had received different cumulative doses of AONs. To study dystrophin levels in the AON-treated mice, total protein was isolated from gastrocnemius muscles (Fig. 4C). Dystrophin levels were low and varied between 0.12% and 0.66% and did not correlate with survival (*R*^2^^=^ 0.1073, *P* = 0.29).

## Discussion

We report, in this study, the results of nonclinical experiments to investigate questions that were raised by clinical trials testing exon skipping AONs in DMD patients. First, we evaluated the effect of age on treatment response. While we did not see a difference in AON uptake and exon skipping levels in young and older *mdx*/BL10 mice, we did observe a positive functional effect of AON treatment only in the younger mice, as assessed by the hanging tests. Notably, this is in alignment with observations in DMD patients described in the introduction, where effects on the 6MWD were clearer in younger patients. DMD is a progressive disorder, where function is irreversibly lost with time [47]. Therapies currently in development aim to slow down disease progression and will not bring back muscle tissue that is lost. As such, the muscle quality at the time of intervention will play an important role in how much patients can potentially benefit from exon skipping therapy. Still, while functions lost will not return with therapies currently in development, studies have shown that patients and parents would welcome therapies that slow down the loss of functions that patients still have [48,49].

Our findings that also in mice older animals appear to benefit less functionally than younger mice are surprising, since it is generally assumed that the *mdx*/BL10 model is relatively mildly affected and that the disease stabilizes after early bouts of degeneration and regeneration between 3 and 6 weeks of age [50]. A possible explanation for the difference in response would be that AON uptake is more efficient during these cycles of degeneration and regeneration. However, AON tissue levels were comparable as were exon skipping levels, except for the diaphragm where exon skipping levels were higher for the older mice. As such, increased uptake cannot underlie the difference in functional response. We did not observe an effect of AON treatment on muscle strength, while the younger mice were stronger than older mice. Lacking functional data for the diaphragm, we do not know whether the higher exon skip levels in diaphragm of older mice improve respiratory function. CK levels were very variable between different mice, as has been reported before [51]. It is known that CK levels are more increased during degeneration and regeneration cycles in younger *mdx* mice and that exercise and activity have an influence on CK levels in mice and humans. Also, dystrophin levels were not higher in the younger mice, which could otherwise have explained the increased functional performance.

Since young and less severely affected DMD patients were more responsive in the intervention, we assessed whether AON, exon skip, and dystrophin levels would differ in the more severely affected DBA/2J-*mdx* mouse. No clear difference in AON, exon skipping, and dystrophin levels could however be observed between *mdx*/BL10 and D2-*mdx* mice treated with the same dosing regimen. We had speculated that dystrophin levels might be lower in D2-*mdx* mice, given their more severe fibrosis. It is important to note that the mice were treated based on a mg/kg dosing. Since the D2-*mdx* are atrophic, while the *mdx*/BL10 mice are hypertrophic it is possible that the relative dosing of muscle is not comparable.

Upon acute physical exercise, one would expect an increased cardiac output and a considerable shift in fractional output to (skeletal) muscle at the expense of other tissues including liver, kidney and spleen. While this could lead to higher AON exposure in muscle, the actual effect may be limited due to the nature of uptake. Being charged and highly protein bound, the cellular uptake of 2OMePS AONs is less determined by perfusion than by factors such as cell surface protein-binding capacity and endocytic activity. We observed that treadmill running indeed had a small but positive effect on AON uptake, resulting in slightly higher AON levels in skeletal muscles compared with sedentary mice, accompanied by a marginal increase in exon skipping and dystrophin levels. It should be noted that the ability of the dystrophic muscle to regulate its blood supply was found to be impaired both in *mdx* mice and DMD patients [52,53], thus the treadmill running may not have had the desired effect. Furthermore, a similar increase in AON levels was seen in nonmuscular tissues. Potentially, renal blood flow and clearance decreased with exercise, thereby increasing tissue exposure in general, although even kidney concentrations seemed to be increased.

Since life expectancy of *mdx*/BL10 mice is only slightly decreased, it is not possible to assess whether AON treatment and exon skipping has an effect on survival in this model. Generally, the *mdx*-*utrn*−/− mouse is used for these studies, as in these mice the additional lack of the dystrophin homolog utrophin underlies a very severe phenotype characterized by severe scoliosis and with death occurring before 12 weeks of age. In these very fragile mice rescue is only possible when intervention is initiated early [54]. Thus far, increased survival has only been reported for *mdx*-*utrn*−/− mice treated with modified AONs able to induce very high exon skipping and dystrophin levels (>40% of wild type) [54–56]. Using a model for skewed X-inactivation, we have previously shown that very low levels of dystrophin (<4%) are able to significantly increase survival [40]. However, in these mice dystrophin was present from birth. In this study, we show, for the first time, that dystrophin restoration at very low levels by intervention starting 1 week after birth significantly improves survival from on average 70–97 days. This is a promising result that hopefully will be translatable to the human situation.

A limitation of our study is the lack of functional analyses on the diaphragm. Especially in the *mdx-utrn−/−* model in which scoliosis and impaired respiratory function play an important role in premature death, it would have been valuable to study whether the increased survival resulted from improved respiratory function. Future studies should be undertaken to shed more light on this. Another limitation is that the studies described here only included 2OMePS AON treatment. It would be worthwhile to study whether PMO treatment would result in a similar benefits, given their different pharmacokinetics and tissue distribution characteristics [24].

Even though eteplirsen is now approved, it is clear that there is room for improvement, because dystrophin levels after treatment are currently very low. While even low levels can have an impact on function and survival, it is anticipated that the therapeutic benefit will be larger with higher levels of dystrophin restoration, as also underlined by animal studies [40,46]. Toward improving exon skipping for DMD, several chemical modifications are being evaluated in nonclinical studies. The tricyclo DNA with a phosphorothioate backbone showed promising results in the *mdx* and *mdx*-*utrn*−/− models, where intravenous injection resulted in exon skipping and dystrophin restoration not only in skeletal muscle but also heart and brain and increased survival in the *mdx*-*utrn*−/− model [56]. Notably, with PMO and 2OMePS efficiency in heart is generally poor, while no effect is observed in brain, since these AONs do not cross the blood–brain barrier. In addition, peptide conjugates to improve delivery and/or muscle homing are under investigation [57–61]. Due to its uncharged nature, it is relatively straightforward to conjugate peptides to the PMO chemistry. It has been shown that conjugating positively charged, arginine-rich peptides to PMOs results in very good uptake in skeletal muscle and heart and high levels of exon skipping and dystrophin restoration in *mdx* mice [57–61] and increased survival in the *mdx*-*utrn*−/− mice [54,55]. Conjugation of peptides to the anionic 2OMePS AONs is more cumbersome. However, conjugation of charge neutral muscle-homing peptides to 2OMePS resulted in increased uptake and exon skipping levels in muscle and heart in the *mdx* model [62,63].

While these new developments are promising, translation to clinical trial phases is not guaranteed. First, upscaling of manufacturing at clinical grade is required to allow testing in humans. Since skeletal muscle is so abundant, relatively high systemic doses will be required. Furthermore, extensive safety tests will have to be conducted for the new chemistries. Finally, while studies in mouse models may be promising, it is possible that efficiency in humans will be lower or even absent.

In summary, in this study we showed that, in line with clinical observations with exon 51 targeting AONs, early intervention with 2OMePS AON treatment in the *mdx*/BL10 mouse model lead to a functional benefit despite low levels of exon skip and dystrophin, whereas a similar effect on exon skip and dystrophin was not accompanied by an apparent functional benefit upon intervention in older mice. The more severe pathology of the D2-*mdx* model does not alter treatment efficiency. Treadmill running seems to slightly increase AON uptake and dystrophin levels in *mdx*/BL10 mice. Furthermore, early intervention with 2OMePS AON treatment significantly increased survival for the *mdx*-*utrn*-−/− mouse model.
